# Evaluating methods for quantitative olfactory assessment: a comparative longitudinal analysis of Sniffin’ Sticks and alternative tools

**DOI:** 10.1093/chemse/bjag012

**Published:** 2026-05-06

**Authors:** Parvaneh Parvin, Valentina Parma, Birgit van Dijk, Sanne Boesveldt

**Affiliations:** Department of Human Nutrition and Health, Wageningen University & Research, Stippeneng 4, Wageningen 6708 WE, The Netherlands; Monell Chemical Senses Center, 3500 Market St., Philadelphia, PA 19104, United States; Department of Otolaryngology, Head and Neck Surgery, University of Pennsylvania, 3400 Spruce Street, Philadelphia, PA 19104, United States; Department of Human Nutrition and Health, Wageningen University & Research, Stippeneng 4, Wageningen 6708 WE, The Netherlands; Department of Human Nutrition and Health, Wageningen University & Research, Stippeneng 4, Wageningen 6708 WE, The Netherlands

**Keywords:** anosmia, olfactory dysfunction, psychophysical tests, smell loss, self-report

## Abstract

Anosmia and hyposmia, referring to total or partial smell loss, respectively, affect 3% to 20% of people. The Sniffin’ Sticks Extended Test, assessing odor threshold, discrimination, and identification (TDI), is a well-validated tool widely used in European and US clinics. However, its time and resource demands limit routine use. During the COVID-19 pandemic, several rapid smell tests were developed, yet their classification performance and agreement with established psychophysical measures remain underexplored. We compared 4 alternatives—the Visual Analog Scale for self-rated smell ability (VAS), the smell section of the Appetite, Hunger, and Sensory Perception questionnaire (AHSP), the Global Consortium for Chemosensory Research Smell Check (GCCR-Check), and the SCENTinel rapid smell test—against TDI scores in a longitudinal cohort of 96 adults (77 female patients; age 46.6 ± 10.5 yr) with post-COVID-19 smell dysfunction, assessed up to 5 times over 12 mo. Analyses used Generalized Estimating Equations for repeated measures, Bland–Altman plots for agreement and bias, and area under the receiver operating characteristic (AUC) curves for classification. No tool dominated across all TDI-defined categories. SCENTinel showed robust performance for anosmia (0.81) with the most balanced sensitivity–specificity trade-off among rapid tests, while GCCR-Check achieved the highest AUC for anosmia (0.88) and VAS best identified normosmia (0.73). Agreement analyses revealed systematic biases in self-report and rapid psychophysical tests. Rapid tools reliably detect anosmia, while classification performance decreases near diagnostic boundaries, particularly for normosmia. Combining brief self-report and short psychophysical measures may improve accuracy while maintaining feasibility for clinical and large-scale screening.

## 1. Introduction

Sensory health is public health. Human olfaction provides critical information for health and well-being (Boesveldt and Parma 2021), and its dysfunction has been linked to a plethora of medical conditions, as recently noted in a systematic review by Leon et al. (2024). For example, olfactory dysfunction has been documented in various neurodegenerative disorders, including Alzheimer's disease (Viguera et al. 2018) and Parkinson's disease (Bagherieh et al. 2023), as well as in unipolar depression (Kazour et al. 2020), epilepsy (Khurshid et al. 2019), autism (Gerkin et al. 2021), and anxiety disorders (Chen et al. 2021). Beyond disease-specific links, smell dysfunction is also associated with psychological burden (Croy et al. 2014b; Alam et al. 2019), altered dietary behavior (Kershaw and Mattes 2018), cognitive decline, and even increased mortality risk (Ekstr¨om et al. 2017). These implications were amplified by the COVID-19 pandemic, which made olfactory dysfunction a highly visible marker of systemic illness. Estimates of smell disorders worldwide before COVID-19 suggest that between 1.4% and 24.5% of the population suffers from olfactory dysfunction, whether self-reported (1.4% to 15.3%) or psychophysically assessed (2.7% to 24.5%) (Grosmaitre et al. 2009). COVID-19 has likely increased this number, as at least 70% of infected individuals experience some degree of olfactory dysfunction, ranging from mild and temporary to severe and long term (Vaira et al. 2022).

Taken together, this evidence points to the need for routine and standardized olfactory evaluation. However, outcome measures in this field have historically lacked agreement across tests. A recent initiative proposed a Core Outcome Set (COS), defined as standardized sets of outcomes that should be measured/reported as determined by consensus, for clinical trials in olfactory disorders (Philpott et al. 2023). In parallel, expert recommendations from the Ear, Nose, and Throat community emphasize the importance of using validated psychophysical tools—particularly those assessing odor threshold, discrimination, and identification—to ensure a clinically meaningful assessment (Whitcroft et al. 2023). Aligning new or existing smell tests with these guidelines is essential to improve both the detection and longitudinal monitoring of olfactory disorders, ultimately improving the care of patients with smell dysfunction.

Despite the availability of commercial psychophysical smell tests, their use remains rare in both clinical settings and population-wide screenings. The Sniffin’ Sticks Extended test (Haehner et al. 2009), which assesses odor threshold, discrimination, and identification, and the University of Pennsylvania Smell Identification Test (Doty et al. 1984), which measures odor identification alone, are both well-validated psychophysical tools for classifying anosmia (complete loss of smell), hyposmia (partial smell loss), and normosmia (normal sense of smell). However, their high cost, reliance on trained personnel, and lengthy, cognitively demanding administration limit scalability and may introduce fatigue-related bias, reinforcing the need for shorter, low-burden alternatives. As a result, a variety of alternative methods have emerged to meet the growing demand for accessible (easy-to-use) olfactory assessment (Li et al. 2024), particularly accelerated by the COVID-19 pandemic. For example, the SCENTinel test includes subcomponents for odor detection, intensity, and identification, and can be completed without supervision in under 5 min. Similarly, the Global Consortium for Chemosensory Research created the GCCR Smell-&-Taste-Check (GCCR-Check), which asks participants to rate the perceived odor intensity of familiar household items across repeated sessions. For a comprehensive overview of other available assessment tools, see Parma and Boesveldt (2021).

In contrast to psychophysical approaches, self-report tools rely on introspective judgments and vary in how they frame the question of olfactory ability. Simple questions such as “Did you notice a sudden change in your sense of smell?” or “Rate your smell on a scale of 0 to 100” using a Visual Analog Scale (VAS) have proven not only diagnostically informative but also effective in raising public awareness of olfactory health (Parma and Boesveldt 2021). Their use may improve early detection, support longitudinal tracking, and help stratify patients at risk for neurodegenerative disease or persistent sensory dysfunction. Although self-report tools are often criticized for their limited sensitivity—since individuals may not always detect gradual olfactory loss (Adams et al. 2017; Boesveldt et al. 2017)—subjective ratings can still provide clinically meaningful information. For instance, population-based studies have shown that self-assessed olfactory impairment is associated with poorer cognitive performance (Yahiaoui-Doktor et al. 2019) and may independently predict conversion to dementia over time (Stanciu et al. 2014). These findings suggest that self-report and psychophysical tests may capture complementary aspects of olfactory dysfunction, each offering a distinct value depending on the clinical or research context.

This study aims to evaluate the classification performance of anosmia and normosmia of 4 olfactory tests—VAS, smell items in the “Appetite, Hunger, Sensory Perception” (AHSP) questionnaire, SCENTinel, and GCCR-Check—relative to widely validated and commonly used Sniffin’ Sticks, using data from a longitudinal cohort of COVID-19 patients with persistent (>1 mo) olfactory dysfunction. Based on the underlying design and measurement characteristics of each test, we expect different levels of agreement with the TDI score of the Sniffin’ Sticks Extended Test. We expect the psychophysical SCENTinel test to show the highest agreement, as it (similarly) targets multiple dimensions of olfactory function; The GCCR-Check, despite involving smelling odor stimuli (aka household items), lacks stimulus control and is thus expected to show moderate agreement. In contrast, we hypothesized that self-report tools (AHSP and VAS) would show lower agreement due to their reliance on subjective perception and spontaneous awareness and would be prone to recall bias. Across all tests, we expect greater accuracy in classifying anosmia than normosmia, given the clearer sensory boundary in total loss.

## 2. Method

### 2.1 Study design

Participants in this study were part of the COVORTS cohort (COVid cohORT on Smell loss), which has been previously introduced in Boesveldt et al. (2024) and van Dijk et al. (2025). This cohort included patients between 18 and 60 yr with persistent (*>* 1 mo) self-reported olfactory dysfunction after recent (*<*3 mo) COVID-19 infection, confirmed with a positive PCR test, or a positive SARS-CoV-2-antigen self-test. In the COVORTS study, patients were followed longitudinally for 12 mo. They were visited at home by the researchers every 3 mo for extensive psychophysical testing of olfactory functioning using Sniffin’ Sticks Extended test. Time points for these measurements were:

- T1: performed as soon as possible after inclusion (baseline);
- T4: 3 mo after baseline;
- T7: 6 mo after baseline;
- T10: 9 mo after baseline;
- T13: 12 mo after baseline.

At the baseline measurement, patients were asked to report their date of birth and sex. Moreover, participants filled out monthly online questionnaires on olfactory functioning, including self-reports on VAS (Parma et al. 2020), the smell section of AHSP (Nguyen et al. 2023), the GCCR-Check (Nguyen et al. 2023), and SCENTinel 1.0 (Parma et al. 2021), a multifunction rapid smell test.

A subgroup of patients (referred to as MRI patients in Table 1) in this study were part of the COVORTS-MRI cohort (*N* = 21). Within this subgroup, patients recruited within 6 mo of their positive COVID-19 test underwent 2 assessments spaced 6 mo apart (*n* = 2). If patients were recruited later than 6 mo after infection, they underwent only 1 measurement. The MRI subgroup of the patients performed Sniffin’ Sticks, VAS, AHSP, and Scentinel 1.0. GCCR-Check data were available for 76 participants, while all other tests also had 21 more participants (*N* = 87). Patients’ characteristics at T1 can be found in Table 1. This study was approved by a regional medical research ethics committee (28–09-2021, file NL77954.091.21) and carried out in compliance with relevant laws and institutional guidelines and in accordance with The Code of Ethics of the World Medical Association (Declaration of Helsinki) for experiments involving humans. All participants provided written consent.

**Table 1:** Participant characteristics.

Characteristic	COVORTS participants	MRI participants
Age (yr, mean ± SD, range)	46.6 ± 10.5 (18 to 60)	46.8 ± 10.5 (19 to 58)
Sex (female/male)	63/13	15/6
BMI (kg/m^2^, mean \pm SD, range)	25.6 ± 4.4 (19.3 to 37.9)	25.8 ± 3.6 (18.3 to 33.5)
Days since known SARS-CoV-2 infection at T1	82.9 ± 22.0 (37 to 128)	835.4 ± 474.0 (76 to 1,447)
Days since known SARS-CoV-2 infection at T13	452.9 ± 21.2 (405.0 to 495.0)	307.0 ± 26.9 (288.0 to 326.0)
Vaccination status (yes/no)	56 (86.6%)/13 (13.4%)	19 (90.5%)/2 (9.5%)

### 2.2 Olfactory measurement tools

This study included 5 olfactory tests, each differing in format, administration, and intended use. Table 2 summarizes the main features of each test, including stimulus type, delivery method, scoring format, and approximate duration.

**Table 2:** Comparison of olfactory tests included in this study.

Feature	VAS	AHSP (Smell section)	GCCR-Check (Home Test)	SCENTinel	Sniffin’ Sticks
**Odor Source**	None	None	Real household items (chosen by participant)	Standardized odor patch	Odor-filled pens
**Delivery**	Online survey	Paper/Online questionnaire	Self-administered at home	Lift’nSmell® card	In-person by trained personnel
**Measurement Type**	Self-report	Self-report	Semi-psychophysical	Psychophysical	Psychophysical
**Control over Stimuli**	N/A	N/A	Low (item-dependent)	High (fixed odor and task)	High (fixed odor and task)
**Test Format**	1 item; 100-point VAS	6 items; Likert-scale (1 to 5)	4 items; 100-point VAS intensity ratings	3 items; Detection (binary), Intensity (0 to 100) and Identification (binary)	48 items; Threshold and Discrimination (triplets), Identification (4-AFC)
**Score Type**	Single continuous score (0 to 100)	Composite score (6 to 30)	Composite average (VAS 0 to 100)	Composite score (across 3 subtests)	Composite sum score (0 to 48)
**Time to Complete**	∼1 min	2 to 3 min	5 min	5 min	30 to 40 min

4-AFC = 4-Alternative Forced Choice, where participants select 1 of 4 options.

#### 2.2.1 Sniffin’ Sticks extended test (TDI, widely validated and commonly used in European research)

Psychophysical olfactory functioning was evaluated with the Sniffin’ Sticks test battery (Burghart, Wedel, Germany) (Kobal et al. 1996). The test consists of 3 parts: odor threshold, odor discrimination, and odor identification. For this study, the extended Sniffin’ Sticks identification set (32 odors) (Haehner et al. 2009). Out of the 32, we created several balanced sets of 16 items (in terms of difficulty), and randomized those across participants and test sessions, to prevent learning effects. Likewise, for the discrimination test, the order of triplets was randomized across patients and test sessions. The 3 subtest scores were summed to yield a composite Sniffin’ Sticks score (TDI, range: 1 to 48), with higher values indicating better olfactory function. Based on established cutoffs (Oleszkiewicz et al. 2019), TDI scores *≤*16.25 indicate anosmia, scores between 16.25 and 30.75 indicate hyposmia, and scores *≥* 30.75 indicate normosmia.

#### 2.2.2 Visual analog scale

Similar to Parma et al. (2020), subjective olfactory ability was measured on a VAS. Patients were asked to quantify their current olfactory ability on a VAS scale ranging from 0 to 100 anchored by “no sense of smell” to “excellent smell”.

#### 2.2.3 Appetite, hunger, and sensory perception questionnaire

Subjective olfactory and gustatory function was also measured using the AHSP questionnaire (Mathey et al. 2001). The “Smell” section of this questionnaire consists of 6 items assessing current and pre-illness olfactory functioning. The responses are given on a 5-point Likert scale, resulting in a score range of 6 to 30 for the smell subscale, with higher scores indicating better olfactory function. An example item is: “In the past, most things used to smell …”, with response options ranging from “much better than nowadays” to “much worse than nowadays.”

#### 2.2.4 The GCCR smell-&-taste-check (GCCR-check)

The home test of the GCCR-Check was used to allow patients to self-assess their olfactory abilities (Nguyen et al. 2023). In this home test, patients rate the intensity of smell sensations of 4 household items (chosen from a list of 42 items, e.g. coffee, vinegar, peanut butter) on a VAS scale ranging from 0 to 100, anchored “no smell” to “very intense”. Intensity ratings across the 4 products were averaged into 1 score.

#### 2.2.5 SCENTinel

Participants performed SCENTinel, a rapid, self-administered, multifunctional smell test, at home (Parma et al. 2021). The test comprises 3 core subtests presented in the following order: odor detection (SCENT-D), a binary task; odor intensity (SCENT-Int), a continuous rating; and odor identification (SCENT-I), also a binary task. Based on predefined criteria that combine performance across these subtests, a composite binary outcome called the Overall Score (SCENT-O) is generated, indicating whether the participant passes or fails the test (Normosmia vs. Anosmia). In this study, the SCENT-O Score was used as the primary classification outcome, while individual subtests were analyzed separately to explore their relative classification contributions. For further details on scoring logic and validation, see Hunter et al. (2023).

### 2.3 Data analysis

This study draws on 4 longitudinal datasets evaluating the performance of 4 olfactory tests (VAS, AHSP, GCCR-Check, and SCENTinel) against the Sniffin’ Sticks TDI score, considered a well-established method in psychophysical olfactory assessment. All datasets include demographic variables such as age, sex, and BMI.

Most participants (N = 76) contributed repeated measurements across 5 time points (T1, T4, T7, T10, T13), while a smaller MRI subgroup (N = 21) provided only 1 or 2 data points depending on the timing of recruitment. One participant was excluded from all datasets due to a COVID-19 reinfection. To retain as much usable information as possible, missing data were handled using pairwise deletion. Participants with incomplete data at certain time points remained in the dataset; only their missing observations were excluded when reshaping the data from wide to long format. To compare the performance of the 4 olfactory tests (AHSP, VAS, GCCR-Check, and SCENTinel) to the Sniffin’ Sticks Extended Test (TDI scores), we conducted a series of statistical analyses tailored to the structure and properties of each dataset (i.e. binary vs. continuous metrics, sample size).

#### 2.3.1 Correlation and agreement

To assess the relationship between each alternative test and TDI scores across time points, Generalized Estimating Equations (GEE) models were used. This method accounts for the longitudinal structure of the data and within-subject correlations. For each test, we modeled TDI as the dependent variable using a Gaussian family, with each test score as the main predictor. To enable comparison of effect sizes across different olfactory tests, each continuous predictor (VAS, AHSP, GCCR-Check, and SCENTinel -Int) was standardized (z-score transformation) before fitting a GEE model. This ensured that all predictors were on a common scale, allowing more interpretable model coefficients despite differences in measurement range and units. Binary predictors (SCENT-D and SCENT-I) were coded as 0 and 1, with model estimates reflecting the mean difference in TDI between response categories. Coefficients from binary and continuous predictors are not directly comparable in magnitude (Gelman and Hill 2007). For VAS, AHSP, GCCR-Check, and SCENT-Int, overall agreement with TDI was further evaluated using Bland-Altman analysis. To account for within-subject correlation due to repeated measures, a GEE model with an exchangeable correlation structure was used. Normality was tested using the Shapiro-Wilk test to determine whether percentile-based Limits of Agreement (LoA) were required. As a result, we computed two types of LoA: A GEE-based LoA, calculated using the estimated mean difference and its robust standard error from the GEE model, and a Percentile-Based LoA, derived from the empirical 2.5th and 97.5th percentiles of the observed differences to account for non-normality in the data. No data transformations were applied, as differences were deemed interpretable on the original scale.

#### 2.3.2 Classification performance

To evaluate whether each olfactory test (VAS, AHSP, GCCR-Check, and SCENTinel) could classify individuals into *Anosmia vs. (Hyposmia + Normosmia)* and *Normosmia vs. (Anosmia + Hyposmia)*, we computed Receiver Operating Characteristic (ROC) curves. The area under the curve (AUC) was used as a summary measure of discriminatory ability, with values closer to 1.0 indicating better performance. For all continuous predictors (VAS, AHSP, GCCR-Check, and SCENT-Int), we used GEE with a binomial family to predict the probability of each diagnostic category. Models included Age and Sex as covariates and accounted for repeated measurements using an exchangeable correlation structure (Zeger and Liang 1988). Given the class imbalance in the TDI-based categories (Normosmia: 79, Hyposmia: 207, Anosmia: 29), inverse-frequency weights were applied before model fitting (Van Hulse et al. 2007). The same GEE modeling approach was used for SCENTinel’s binary subcomponents (Odor Detection, Odor Identification, and Overall Score). Although these variables are binary by nature, fitting GEE models allowed us to derive predicted classification probabilities while adjusting for covariates and the longitudinal data structure. This enabled consistent ROC-based evaluation across all test components. To convert predicted probabilities into binary classifications, we determined the optimal threshold for each contrast using the Youden Index (Hsieh et al. 2017), which maximizes the sum of sensitivity and specificity. These thresholds were then used to derive predicted class labels, enabling a direct comparison with the alternative tests with TDI-based diagnostic categories. While AUC reflects overall discrimination, we also visualized predicted probabilities across TDI scores to identify where models succeeded or failed. These plots (in Appendix A, Supplementary Figure A.3) were used to assess whether mis-classifications occurred near clinical diagnostic thresholds, offering insight into model confidence and decision boundaries. Classification performance was evaluated using precision, sensitivity (recall), F1-score, Cohen's Kappa, and percentage agreement (Sokolova and Lapalme 2009). All metrics were computed separately for each binary classification task (i.e. anosmia vs. other, normosmia vs. other) to enable a systematic comparison of diagnostic utility across tests.

We did not report classification performance of the *Hyposmia vs. (Anosmia + Normosmia)*, because merging extreme categories creates an artificial central tendency that could lead to spurious findings and misinterpretation of results.

#### 2.3.3 Pairwise comparison of test performance

To statistically compare the diagnostic performance of the alternative tests, we conducted pairwise comparisons of AUC values using the DeLong method (DeLong et al. 1988), which allows for a nonparametric comparison of correlated ROC curves. This approach accounts for the paired nature of predictions, as each participant completed all tests. Predicted probabilities were extracted from Generalized Estimating Equation (GEE) models for each test, adjusted for age and sex, and used to compute the DeLong test statistics between all test pairs. For each binary classification task (e.g. Anosmia vs. Other), we compared all test combinations (VAS, AHSP, GCCR-Check, and SCENTinel components). To adjust for multiple comparisons within each classification category, we applied False Discovery Rate (FDR) correction using the Benjamini–Hochberg procedure with an alpha level of 0.05.

#### 2.3.4 Software and implementation

All analyses were conducted in Python (v3.12.7) using Jupyter Notebooks (Kluyver et al. 2016). Statistical modeling was performed with statsmodels (Seabold and Perktold 2010), and classification tasks were implemented using scikit-learn (Pedregosa et al. 2011). Visualizations were created with matplotlib and seaborn. GEE were implemented via statsmodels, and ROC-based evaluations, including DeLong's test, were customized using available nonparametric routines.

## 3. Results

### 3.1 Participant demographics and dataset descriptives

Table 3 summarizes participant demographics and test-specific results for each olfactory ability (i.e, anosmia, hyposmia, normosmia). Data from each smell test included repeated measures across 5 time points (T1–T13), and demographic variables such as age, sex, and body mass index. TDI was used as the reference for merging, and the final long-format datasets retained only complete paired observations. The VAS, AHSP, and GCCR-Check datasets showed increasing dropout at later time points, with GCCR-Check missingness distributed equally across odor items. For VAS, 18 entries from 13 participants were intentionally excluded based on low self-reported pre-COVID olfactory ability (VAS *<* 40). SCENTinel was incorporated in the study design at a later time point, and hence has fewer data points.

**Table 3:** Test characteristics for each olfactory measure, including demographic variables and average scores across TDI-defined smell function categories.

Measure	VAS	AHSP	GCCR-Check	SCENT-O (%)
**Number of participants**	96	96	76	74
**Missing data points**	131	113	36	—
**Final data points (long)**	348	366	343	137
**Sex (female/male)**	77/19	77/19	63/13	58/16
**Age (years, M ± SD)**	46.6 ± 10.5	46.6 ± 10.5	46.6 ± 10.5	47.6 ± 9.7
**BMI (kg/m^2^, M ± SD)**	25.6 ± 4.2	25.6 ± 4.2	25.6 ± 4.4	25.9 ± 4.2
**Anosmia**	14.8 ± 18.4	8.7 ± 2.2	17.6 ± 17.9	16.7
**Hyposmia**	40.2 ± 26.7	12.8 ± 4.5	50.6 ± 26.4	64.6
**Normosmia**	58.7 ± 27.9	15.6 ± 4.9	65.9 ± 24.8	86.0

Values in the last 3 rows (anosmia, hyposmia, normosmia) represent mean scores or % correct (for SCENT-O) per TDI category.

Shapiro–Wilk tests indicated statistically significant deviations from normality for all continuous variables (*P*  *<* 0.001). Visual inspection confirmed substantial non-normality for VAS, GCCR-Check, and SCENT-Int scores, which displayed skewed or multimodal distributions. AHSP scores were bounded and discrete, further violating normality assumptions. TDI scores appeared approximately normal upon visual inspection, and the significant result from the Shapiro–Wilk test likely reflects the test's sensitivity to minor deviations in larger samples rather than meaningful non-normality. These results collectively support the use of non-parametric or robust models in all subsequent analyses. Across all tests, mean scores increased with olfactory function as measured psychophysically, showing a clear separation between anosmia, hyposmia, and normosmia groups. This was most pronounced for SCENT-O (16.7% in anosmia vs. 86.0% in normosmia) and GCCR-Check (17.6 in anosmia vs. 65.9 in normosmia), and consistent for VAS and AHSP as well, reflecting strong alignment between subjective or simplified measures and the Sniffin’ Sticks TDI classification.

### 3.2 Correlation between VAS, AHSP, GCCR-check, SCENTinel, and TDI

GEE models using standardized predictors revealed that all alternative test scores were significantly associated with TDI scores (Table 4). Among continuous predictors, the strongest associations were observed for GCCR-Check and SCENT-Int, followed by AHSP and VAS. The SCENT-O Score was also positively associated with TDI, while its binary components (Odor Detection and Identification) showed negative coefficients, indicating lower TDI scores among participants who scored 0 on these subtests. These results confirm that both the psychophysical and the self-administered tools reflect TDI scores to varying degrees, with greater correspondence observed for more psychophysical and intensity-based measures. Note that only continuous predictors were standardized; binary variables reflect group-level contrasts.

**Table 4:** Association between test scores and TDI using GEE models. Predictors were z-standardized where applicable.

Test	Coefficient (β)	SE	95% CI	*P*-value
VAS	2.44	0.36	[1.74, 3.16]	<0.001
AHSP	2.50	0.38	[1.74, 3.26]	<0.001
GCCR-Check	2.69	0.39	[1.91, 3.47]	<0.001
SCENT-O	4.90	1.16	[2.62, 7.19]	<0.001
SCENT-D	−7.76	3.00	[−13.65, −1.88]	0.010
SCENT-Int	3.37	0.74	[1.91, 4.84]	<0.001
SCENT-I	−3.00	1.20	[−5.35, −0.64]	0.013

Coefficients stand for the estimated change in TDI score per 1 SD (for continuous predictors) or mean difference in TDI between response categories (1 vs. 0 for binary SCENTinel components). SE = standard error; CI = confidence interval; SD = standard deviation.

### 3.3 Agreement between VAS, AHSP, GCCR-check, SCENTinel, and TDI (Bland–Altman analysis)

**Figure 1:**
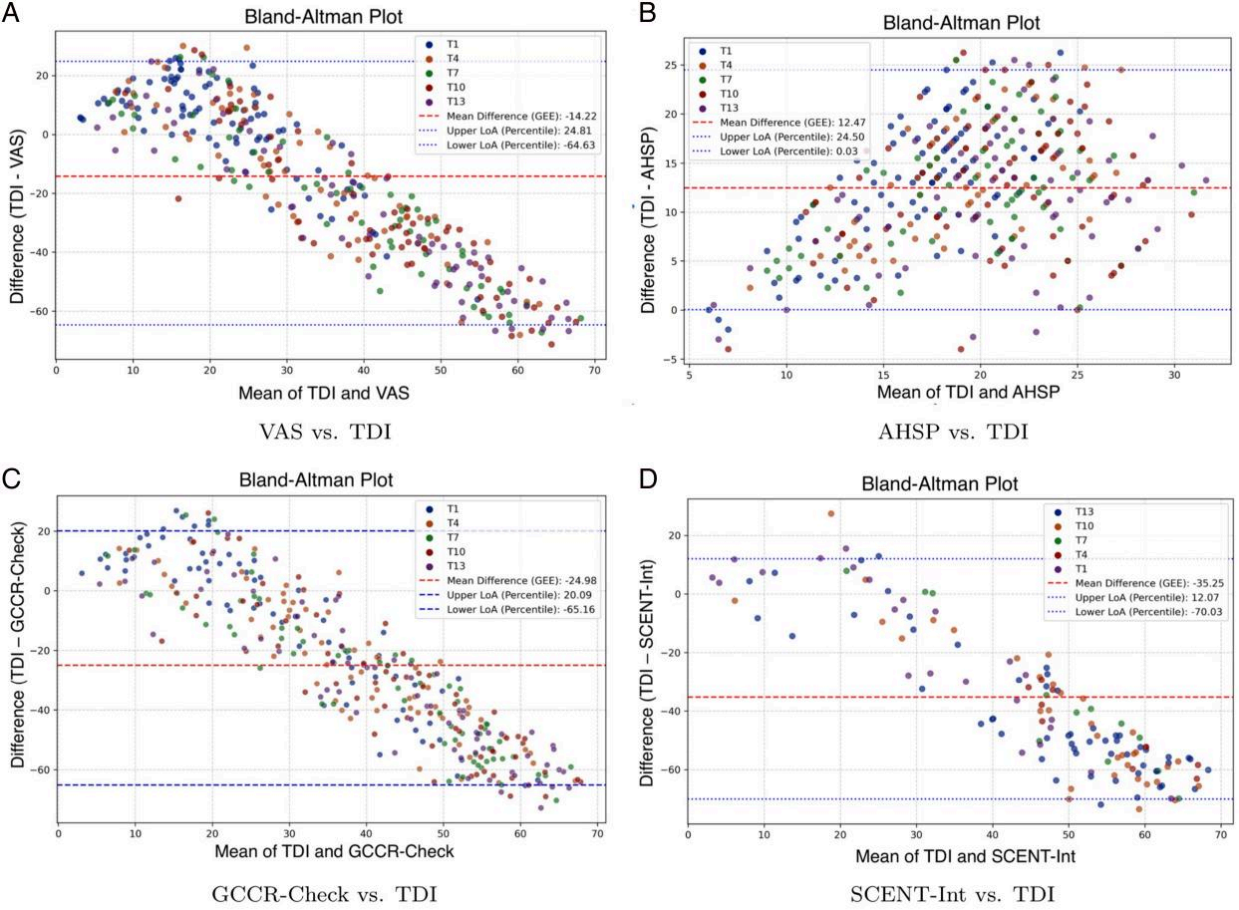
Bland-Altman plots comparing agreement between TDI and alternative olfactory tests across longitudinal time points (T1, T4, T7, T10, T13). Each panel shows the difference between 1 alternative test and TDI (*y*-axis) against the mean of the 2 scores (*x*-axis). Panels show: (a) VAS vs. TDI; (b) AHSP vs. TDI; (c) GCCR-Check vs. TDI; (d) SCENT-Int vs. TDI. Colors correspond to different time points. Dashed lines indicate the mean bias and limits of agreement.

The Bland–Altman analysis revealed systematic differences between alternative tests and the TDI score, with varying levels of agreement and bias (Fig. 1). The **VAS** test exhibited a mean difference of −14.22 (standard error [SE] = 2.12), with limits of agreement (LoA) ranging from −64.63 to 24.81. This indicates a systematic underestimation of olfactory performance relative to TDI, accompanied by substantial individual variability. A proportional bias was confirmed using a GEE model with the mean score as a predictor (*β* = *−*1.54, SE = 0.04, *z* = *−*36.17, *P*  *<* 0.001, 95% confidence interval [CI]: [−1.62, −1.46]), indicating that underestimation increases at higher levels of olfactory ability. In contrast, the **AHSP** test showed a positive mean bias of 12.47 (SE = 0.52), with LoA between 0.03 and 24.50. This test also demonstrated proportional bias, with greater overestimation at lower olfactory ability levels (*β* = 0.51, SE = 0.09, *z* = 5.24, *P*  *<* 0.001, 95% CI: [0.32, 0.70]). The **GCCR-Check** test demonstrated the largest negative mean bias of −24.98, with LoA from −65.16 to 20.09. A strong proportional bias was also detected (*β* = *−*1.50, SE = 0.047, *z* = *−*31.93, *P*  *<* 0.001, 95% CI: [−1.59, −1.41]). For **SCENTinel**, only the SCENT-Int subcomponent was included in the analysis due to its continuous scale. This component yielded a mean difference of −35.25, with LoA between −70.03 and 12.07, reflecting a pronounced tendency to underestimate olfactory performance measured by TDI. A formal test confirmed the presence of proportional bias (*β* = *−*1.41, SE = 0.07, *z* = *−*18.61, *P*  *<* 0.001, 95% CI: [−1.56, −1.26]), consistent with the patterns seen in VAS and GCCR-Check. Although SCENT-Int is not a standalone classification measure, its inclusion highlights the subjective scaling behavior of participants when rating odor intensity.

### 3.4 ROC performance summary

#### 3.4.1 VAS

In the anosmia model, VAS ratings were a significant negative predictor (*β* = *−*0.06, *P*  *<* 0.001), indicating that lower VAS ratings were associated with a higher likelihood of anosmia. Sex was also significant (*β* = *−*1.37, *P* = 0.019), with male patients less likely to be classified as anosmic based on VAS ratings. Additionally, VAS was a strong positive predictor of normosmia (*β* = 0.04, *P*  *<* 0.001), and age showed a small negative effect (*β* = *−*0.04, *P* = 0.027), suggesting that older individuals tend to be associated with reduced likelihood of normosmia.

#### 3.4.2 AHSP

Lower AHSP ratings were significantly associated with anosmia (*β* = *−*0.45, *P*  *<* 0.001), and sex was marginally significant (*β* = *−*1.16, *P* = 0.051), suggesting females were more likely to be classified as anosmic at equivalent AHSP levels. For normosmia, higher AHSP ratings (*β* = 0.22, *P*  *<* 0.001) and younger age (*β* = *−*0.04, *P* = 0.036) were significant positive predictors indicating that younger individuals were more likely to be classified as normosmic.

#### 3.4.3 GCCR-check

GCCR-Check intensity ratings were significantly predictive across diagnostic categories. Lower GCCR-Check ratings were associated with anosmia (*β* = *−*0.08, *P*  *<* 0.001), and higher ratings predicted normosmia (*β* = 0.038, *P*  *<* 0.001). Sex was a significant predictor only in the anosmia model (*β* = *−*1.84, *P* = 0.006), indicating lower odds of being classified as anosmic for males. Age was not significant in the anosmia or hyposmia models, but showed a significant negative association in the normosmia model (*β* = *−*0.035, *P* = 0.043), indicating that older participants were less likely to be classified as normosmic.

#### 3.4.4 SCENTinel

**Figure 2:**
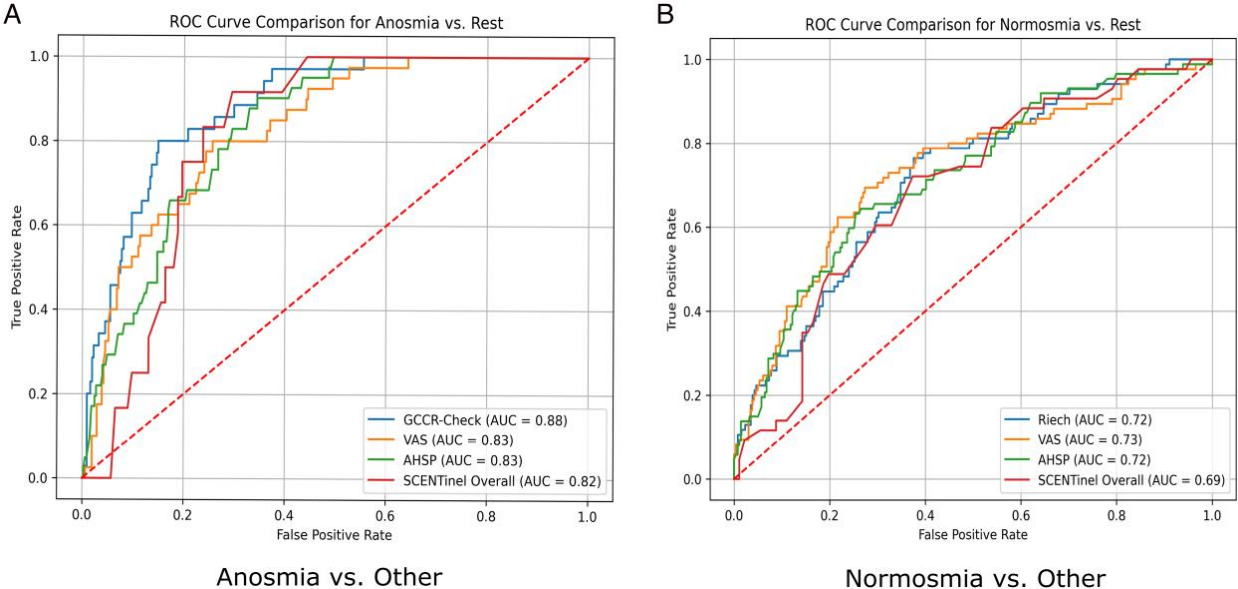
ROC curve comparisons across diagnostic categories. Each panel shows test performance (True Positive Rate vs. False Positive Rate) across thresholds, with AUC values indicated in the legend. (a) Anosmia vs. Other; (b) Normosmia vs. Other.

The SCENT-O (binary) was a strong predictor of both anosmia (*β* = *−*2.87, *P*  *<* 0.001) and normosmia (*β* = 2.26, *P*  *<* 0.001). Among SCENTinel components, SCENT-Int (continuous) was predictive across models: it was negatively associated with anosmia (*β* = *−*0.06, *P*  *<* 0.001) and normosmia (*β* = 0.03, *P*  *<* 0.001). For SCENT-D (binary), those who correctly detected the odor were significantly less likely to be anosmic (*β* = 3.87, *P*  *<* 0.001) and more likely to be normosmic (*β* = *−*3.05, *P* = 0.015). SCENT-I (binary) showed limited predictive value, with only 1 significant association: individuals who correctly identified the odor were more likely to be normosmic (*β* = *−*1.42, *P* = 0.015). No consistent age effects emerged across the models. However, sex significantly predicted anosmia: males were less likely to be anosmic than females (*β* = *−*1.37, *P* = 0.041), while no significant associations were observed for normosmia. To assess classification performance, we calculated AUCs from predicted probabilities derived via GEE models for each test and category (Table 5) as well as the cutoffs—those points at which the sum of sensitivity and specificity are maximized. The ROC curves are represented in Fig. 2.

**Table 5:** Classification performance of olfactory tests across categories.

Test	Anosmia vs. other				Normosmia vs. other			
	AUC	Threshold	Sens	Spec	AUC	Threshold	Sens	Spec
VAS	0.83	0.42	0.80	0.74	0.73	41.5	0.77	0.66
AHSP	0.83	0.30	0.90	0.70	0.72	42.5	0.71	0.68
GCCR-Check	0.88	0.50	0.80	0.85	0.72	0.37	0.76	0.52
SCENT-O	0.81	0.15	0.91	0.70	0.70	0.64	0.72	0.62
SCENT-D	0.81	0.35	0.66	0.84	0.64	0.35	0.74	0.52
SCENT-Int	0.91	0.34	0.91	0.82	0.68	0.37	0.74	0.57
SCENT-I	0.60	0.37	0.41	0.80	0.65	0.34	0.76	0.51

AUC = area under the receiver operating characteristic curve; Threshold = optimal threshold; Sens = sensitivity; Spec = specificity; GEE = generalized estimating equations.

For identifying anosmic individuals, GCCR-Check yielded the highest AUC (0.88), followed by VAS and AHSP (0.83), and SCENT-O (0.81). While GCCR-Check numerically outperformed the other tests, only the comparison between VAS and GCCR-Check reached statistical significance after FDR correction (*P*_adj_  *<* 0.001), indicating that GCCR-Check had significantly better performance than VAS in detecting anosmia. Differences between AHSP and the other main tests were not statistically significant, suggesting comparable diagnostic ability among AHSP, VAS, and SCENT-O.

To further explore SCENTinel's internal structure, we examined its components. For anosmia, SCENT-O outperformed both SCENT-I (AUC = 0.60) and SCENT-D (AUC = 0.81), with statistical significance (*P*_adj_  *<* 0.001 for SCENT-I and *P*_adj_ = 0.001 for SCENT-D). For normosmia, there were no significant differences between SCENT-O (AUC = 0.69) and its sub-components (Detection, Intensity, Identification), all of which performed similarly (*P*_adj_  *>* 0.15). These component-wise findings provide helpful insight into the classification contributions of SCENTinel's subtests but reinforce the importance of interpreting them in the context of the full SCENT-O score. Full pairwise comparisons are reported in Appendix A.4, Supplementary Table A.9.

Moving beyond overall discrimination, the classification metrics (see Table 6) revealed additional insights into each test's classification performance. All tests demonstrated the strongest performance in identifying anosmia, with the highest F1-scores observed for GCCR-Check (F1 = 0.51, Kappa = 0.44, 85% agreement). Among SCENTinel's subcomponents, SCENT-Int also achieved a strong F1-score (0.50, Kappa = 0.43, 84% agreement) comparable to GCCR-Check and higher than SCENT-O, while SCENT-D and SCENT-I performed less consistently. For normosmia, classification performance was moderate across tests. SCENT-O again showed the highest F1-score (0.57), followed closely by VAS (0.54), GCCR-Check (0.52), and AHSP (0.51). Cohen's Kappa values for SCENT-O and VAS were all above 0.26, indicating fair agreement with TDI-defined categories. Among SCENTinel subcomponents, SCENT-D and SCENT-I performed comparably (F1 = 0.54 and 0.55, respectively), but did not outperform the composite SCENT-O score. In addition, the confusion matrices for each test across categories (anosmia and normosmia) are provided in Appendix A, Supplementary Figure A.8 to give further insight into the model's classification behavior, particularly regarding false positives and false negatives.

**Table 6:** Classification metrics, Cohen's Kappa, and Percentage Agreement for each test across diagnostic categories.

	Anosmia vs. Other					Normosmia vs. Other				
Test	Precision	Recall	F1-score	Kappa	% Agree	Precision	Recall	F1-score	Kappa	% Agree
VAS	0.29	0.80	0.42	0.31	75%	0.45	0.69	0.55	0.35	72%
AHSP	0.25	0.90	0.39	0.26	68%	0.43	0.64	0.52	0.32	71%
GCCR-Check	0.38	0.80	0.51	0.44	85%	0.40	0.77	0.53	0.30	66%
SCENT-O	0.92	0.70	0.37	0.27	72%	0.72	0.63	0.57	0.31	66%
SCENT-D	0.67	0.84	0.41	0.33	83%	0.74	0.53	0.54	0.23	60%
SCENT-Int	0.34	0.92	0.50	0.42	84%	0.45	0.74	0.56	0.27	63%
SCENT-I	0.42	0.80	0.24	0.13	77%	0.77	0.52	0.55	0.23	60%

Precision, Recall (Aka Sensitivity), and F1-score are standard classification metrics. Support = the number of true positive class cases. % Agree = overall classification agreement. Kappa = Cohen's Kappa.

To better understand the distribution of classification errors across the clinical spectrum, we visualized the predicted probabilities by category (anosmia, normosmia) against the corresponding TDI scores for all 4 tests (VAS, AHSP, GCCR-Check, and SCENTinel). Each point represents an individual prediction, color-coded by whether the threshold-based classification matched the original TDI-based diagnosis. The vertical lines denote the diagnostic thresholds used to define TDI categories.

Across tests, misclassifications were observed across the full range of TDI scores, though several tests (notably SCENTinel and AHSP) showed a greater concentration of errors near category boundaries (i.e. around TDI = 16.25 and 30.75). In all tests, predictions for anosmia and normosmia exhibited broad probability ranges. Correct classifications were most common at the TDI extremes, with increased misclassification density for intermediate TDI scores. Full visualizations are presented in Supplementary Figure A.3 in Supplementary Appendix A.2.

## 4. Discussion

Olfactory dysfunction is one of the strongest early indicators of COVID-19 and remains a frequent long-term symptom (Biadsee et al. 2021; Bordin et al. 2021; Lechien et al. 2021; Riestra-Ayora et al. 2021; Ferreli et al. 2022; Hintschich et al. 2022; Nikolopoulou and Maltezou 2022; Tan et al. 2022; Boscolo-Rizzo et al. 2023, 2022; CDC, 2024). Detecting it supports patient management by validating symptoms and guiding counseling on nutrition and food safety (Croy et al. 2014a; Ferrulli et al. 2022), and helps identify individuals who may benefit from olfactory training (Hummel et al. 2009; Oleszkiewicz et al. 2018). Because persistent olfactory dysfunction is linked to reduced quality of life, mood disturbances, and elevated risk of later cognitive decline (Sun et al. 2012; Kollndorfer et al. 2017; Dintica et al. 2019; Lee et al. 2024), formal testing remains valuable for follow-up care (Horwitz et al. 2025).

This study compared 4 alternative olfactory tests—VAS, AHSP, GCCR-Check, and SCENTinel—to the widely validated Sniffin’ Sticks Extended test to classify post-COVID-19 olfactory dysfunction. The selection of alternative short tests in this study reflected a pragmatic balance between methodological diversity and feasibility. We aimed to include tools spanning different formats along the continuum from self-report (VAS, AHSP) to semi-psychophysical home ratings (GCCR-Check) and a rapid psychophysical assessment (SCENTinel). These tests were widely used during the COVID-19 pandemic, aligned with the context of our cohort, and were feasible to implement repeatedly in a longitudinal design.

Although none of the tests consistently outperformed the others across all olfactory ability groups, each demonstrated specific strengths. In particular, and in line with expectations, the classification performance was strongest for anosmia across all tests, and normosmia was classified with moderate reliability, especially by self-report tools.

Another possible explanation for these mismatches is the high prevalence of parosmia in post-COVID cohorts (van Dijk et al. 2025). Individuals with parosmia may report severe dysfunction due to qualitative distortions, although the main impact typically concerns odor identification and discrimination, with threshold performance affected only indirectly. This divergence can lead to self-perceived severe loss despite relatively preserved scores on psychophysical tests such as Sniffin’ Sticks.

GCCR-Check and SCENT-O showed the strongest performance for anosmia detection (AUCs *>* 0.80), while VAS was more effective in identifying normosmia, combining relatively high AUC with good sensitivity and specificity. SCENT-Int, although not designed as a standalone diagnostic tool, provided further support for anosmia identification, yielding the highest AUC among all features. Notably, both SCENT-Int and the *intensity* component of GCCR-Check rely on participants’ ratings of the odor intensity—suggesting that intensity-based judgments may offer a robust, scalable signal to detect severe olfactory loss. Its comparative framing (‘now’ vs. “before’) may increase sensitivity to perceived changes in function. Although performance rankings were largely consistent across analyses, a few differences were statistically significant. Notably, the self-assessed GCCR-Check test significantly outperformed the self-report VAS for anosmia detection. In contrast, SCENT-O demonstrated relatively even performance across categories, achieving good detection of both normosmia and anosmia without a marked trade-off between them. These findings underscore that not all numerical differences in AUC values translate into meaningful ability improvements. Statistical significance must be assessed before concluding that 1 test offers a true diagnostic advantage over another.

Demographic variables also influenced classification accuracy, particularly for GCCR-Check and VAS. Age showed a small but consistent negative effect on normosmia classification across the self-report tools (VAS, AHSP), and GCCR-Check, suggesting that older individuals were less likely to be classified as normosmic. This may reflect a tendency to underreport intact olfactory function or normalize gradual decline as part of aging, even when psychophysical scores indicate normosmia (Murphy et al. 2002). These patterns were not observed in SCENTinel, reinforcing that self-perception and response context play a role in age-related bias, and underlying the need for routine smell evaluation, including psychophysical components, even if presented in short form. Sex differences also emerged, especially in anosmia models, where females were more likely to be classified as anosmic at comparable test scores. These effects should be accounted for when interpreting individual test results, especially in clinical contexts.

Beyond accuracy metrics, agreement analyses revealed systematic response biases, meaning that even when tests correctly classified participants on average, individuals often rated themselves in ways that deviated from their actual psychophysical scores. Both VAS and GCCR-Check tended to underestimate smell ability at higher levels, suggesting that individuals with relatively intact function tend to rate themselves with lower ability than what psychophysical testing (Sniffin’ Sticks) reveals. These findings align with population-level data from the NHANES study, which concluded that self-reported olfactory dysfunction was most accurate among individuals with severe loss, but less reliable for identifying individuals with normosmia who often misjudged their olfactory ability—either under- or overestimating their performance relative to their psychophysical test results (Hoffman et al. 2016). Interestingly, SCENT-Int showed similar underestimation bias and wide limits of agreement, suggesting that participants with near-normal olfactory function may still rate odors as weak or ambiguous. Although this can be due to genetic differences leading to specific anosmias, the odors used in SCENTinel seem to be resistant to this potential confounding factor (Hunter et al. 2024).

In contrast, AHSP showed a systematic overestimation of olfactory function among participants with low TDI scores. This may partly reflect the test's design, which asks participants to rate changes relative to pre-illness function—introducing potential recall bias (Schwarz 1999). Despite this, AHSP exhibited narrower limits of agreement, suggesting less variability in responses across individuals. This structured bias likely reflects how the task frames judgment: AHSP prompts comparison with prior ability, potentially heightening sensitivity to perceived change. However, it may also introduce upward bias in anosmic individuals who recall their prior function as much better, leading to overestimation. VAS, by contrast, captures a more immediate, though generic, self-assessment, which might explain its higher specificity in normosmia classification but poorer sensitivity to mild dysfunction. These patterns suggest that the framing of response scales—not just what is asked, but how—is central to understanding variation in self-reported olfactory performance.

From a practical standpoint, each test offers advantages and trade-offs relevant to clinical application. VAS showed the highest specificity for normosmia, suggesting utility for ruling out dysfunction in broad screening. Its speed and simplicity make it well-suited for population-level use, but its susceptibility to underestimation and wide limits of agreement reduce its reliability as a standalone diagnostic tool. Hence, its reliable normosmia classification supports its use for initial triage, rather than definitive diagnosis.

GCCR-Check is a strong candidate for rapid anosmia screening (especially in large-scale or remote settings) due to its high recall, fast self-administration, and strong AUC. However, its reduced sensitivity for normosmia in older adults suggests caution in age-diverse populations, especially when relying on general TDI cutoffs that do not account for normal age-related decline (Oleszkiewicz et al. 2019). As such, GCCR-Check's apparent overclassification of normosmia in older participants may reflect a closer alignment with age-adjusted norms rather than misclassification *per se*. Still, its normosmia performance was on par with other tests, suggesting it remains useful for detecting intact function in accessible, self-administered formats. When classification performance is comparable, as seen between VAS and GCCR-Check for normosmia detection, other factors such as administration time, participant burden, and cost become critical. SCENT-O offered a robust performance, and the most balanced trade-off between sensitivity and specificity across tests, supporting its suitability for rapid screening applications where missing anosmic cases are more critical than occasional false positives. Its structured, psychophysical design ensures standardization across users and objectivity, which may be preferable in clinical contexts where test administration must be consistent across raters or settings.

One promising direction involves combining multiple test formats, such as a self-report question plus a rapid psychophysical component—for example, odor intensity ratings, as used in SCENTinel and GCCR-Check. This approach aligns with what was suggested by Boesveldt et al. (2017) and is further supported by the recent core outcome set for olfactory dysfunction, which recommends including both psychophysical and self-report measures in clinical trials (Philpott et al. 2023). Alternatively, adaptive thresholds (clinical cutoffs) could be developed based on individual factors (e.g. age, baseline function). Incorporating these elements into digital platforms could further improve scalability and sensitivity.

This study benefits from standardized, well-established measures, a well-characterized cohort, and repeated assessments over time and within person. Despite the limited sample size, the repeated-measures design contributed to a larger number of total observations, enhancing statistical power and enabling more robust estimation of classification performance across tests. This design adds robustness to our findings. However, the present study is not free from limitations. The Sniffin’ Sticks was not always performed on the same day as other tests due to logistical constraints, potentially introducing variability in olfactory outcomes. The sample had an uneven category distribution, particularly a low number of anosmic cases in TDI classifications. Importantly, the sample consisted exclusively of individuals with post-COVID-19 olfactory dysfunction, a population in which parosmia is particularly prevalent van Dijk et al. (2025). While Bland-Altman and classification metrics provide complementary perspectives, neither fully captures classification uncertainty, particularly in borderline or fluctuating cases. Future research should explore dynamic models (e.g. latent class or machine learning classifiers) that better handle these classification gray zones. Moreover, while our methods combined robust metrics (i.e. ROC, Bland–Altman, F1-scores), they assume stable test properties across time and individuals. Future research should explore personalized models, for example, adaptive thresholds based on age, time since onset, or baseline function. Digital integration also offers promise: hybrid tests combining self-report and psychophysical components within a mobile or web-based framework could help overcome current trade-offs between speed and precision.

## 5. Conclusion

This study evaluated 4 olfactory tests (VAS, AHSP, GCCR-Check, and SCENTinel) against the Sniffin’ Sticks Extended test for classifying anosmia and normosmia. Each test showed distinct strengths, but none provided a universally optimal solution across all classification categories within the spectrum of olfactory ability. While anosmia was consistently the easiest to detect, classification accuracy decreased near diagnostic boundaries, particularly for normosmia, underscoring known limitations of current assessment tools. To enhance diagnostic accuracy, particularly in borderline cases, we recommend combining a self-report measure with a brief psychophysical component. Such hybrid approaches are not only scalable but also practical for research, clinical, and population-level applications.

## Supplementary Material

bjag012_Supplementary_Data

## Data Availability

Upon project completion, the data presented in this article will be made available in the DANS-Easy repository at https://doi.org/10.17026/LS/YZHHK7.
