# Supplementary material for: Evaluating methods for quantitative olfactory assessment: a comparative longitudinal analysis of Sniffin’ Sticks and alternative tools
Source: Chem Senses. 2026 May 6;51:bjag012. doi: 10.1093/chemse/bjag012 (PMC13214561; doi:10.1093/chemse/bjag012)
Supplement: bjag012_Supplementary_Data [file bjag012_supplementary_data.zip › Appendix_A.docx]

# Appendix A. Supplementary Materials

## Appendix A.1. Raw Correlation analysis

Table A.7: Correlation between alternative tests and TDI scores using raw data and GEE.

| **Test** | **Correlation Coefficient** | **SE** | **95% CI** | **p-value** |
| --- | --- | --- | --- | --- |
| VAS | 0.08 | 0*.*01 | [0.060, 0.109] | *<* 0*.*001 |
| AHSP | 0.52 | 0*.*08 | [0.362, 0.679] | *<* 0*.*001 |
| GCCR-Check | 0.09 | 0*.*01 | [0.068, 0.123] | *<* 0*.*001 |
| SCENT-O | - | - | - | *<* 0*.*001^∗^ |

*Note:* Correlation coefficients were obtained using GEE models with an exchangeable correlation structure. SE = Standard Error, CI = Confidence Interval. GEE = Generalized Estimating Equations

^∗^ Direct correlation coefficient was not computed for SCENTinel due to data structure differences.

##
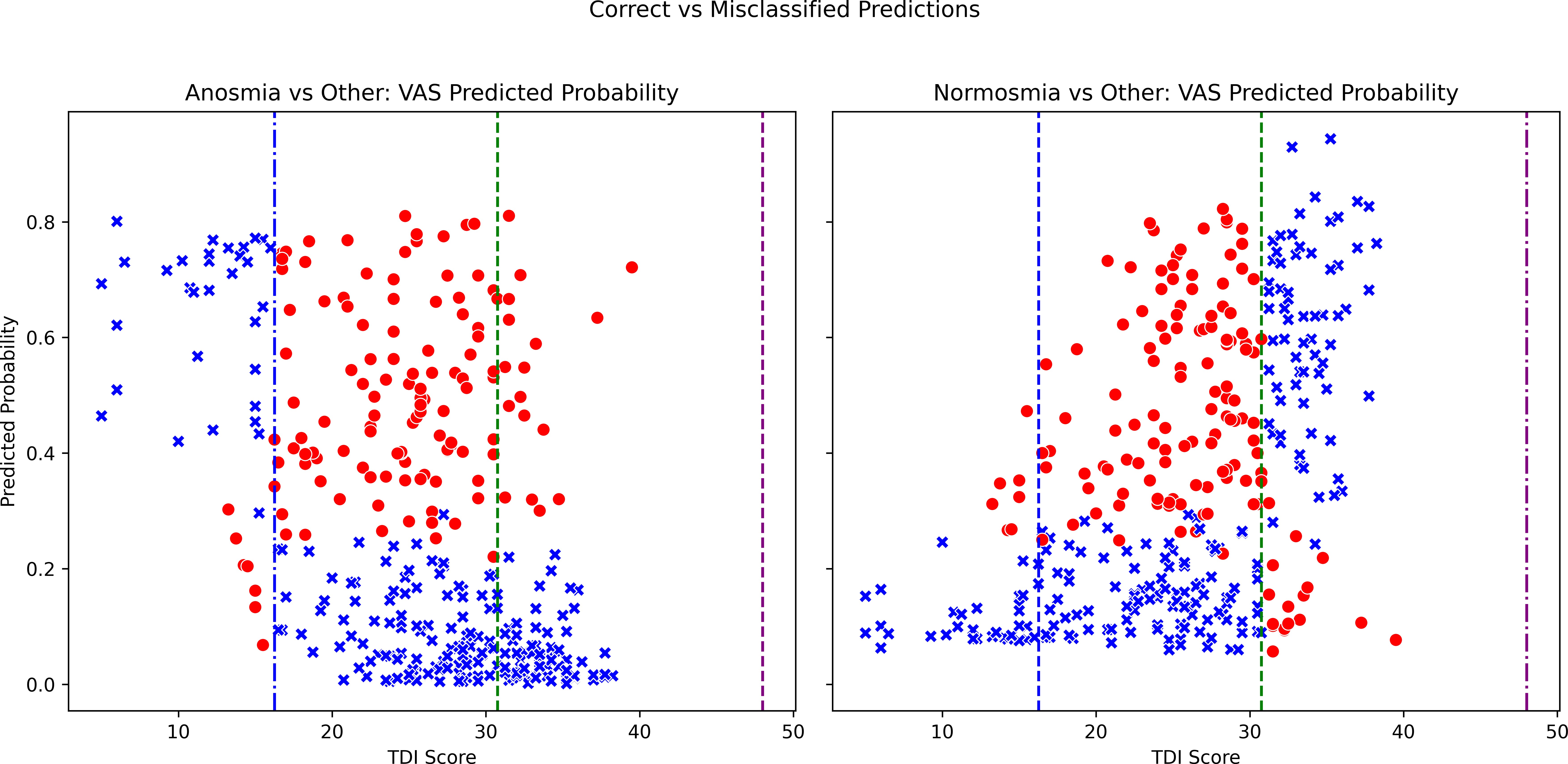
Appendix A.2. Misclassification

*VAS*

- - 1. VAS


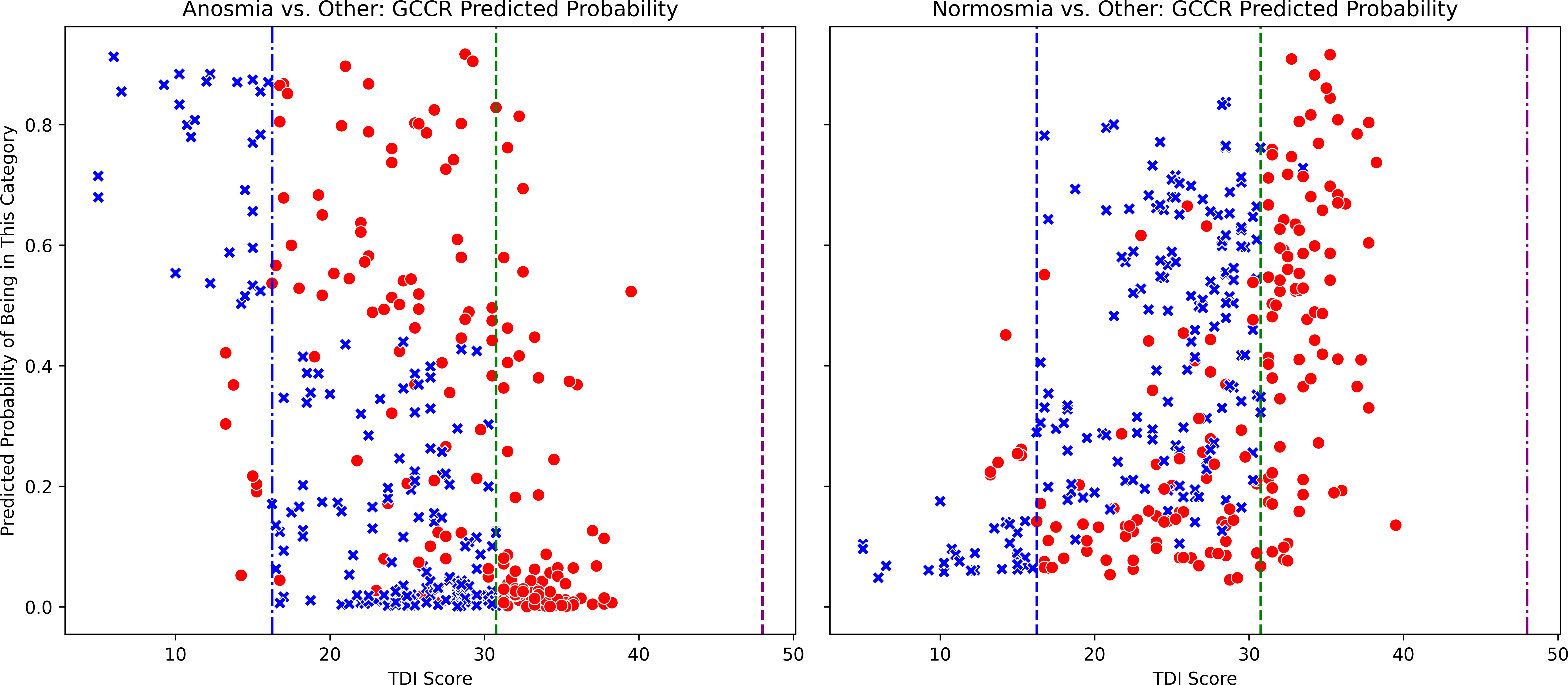


- - 1. GCCR-Check


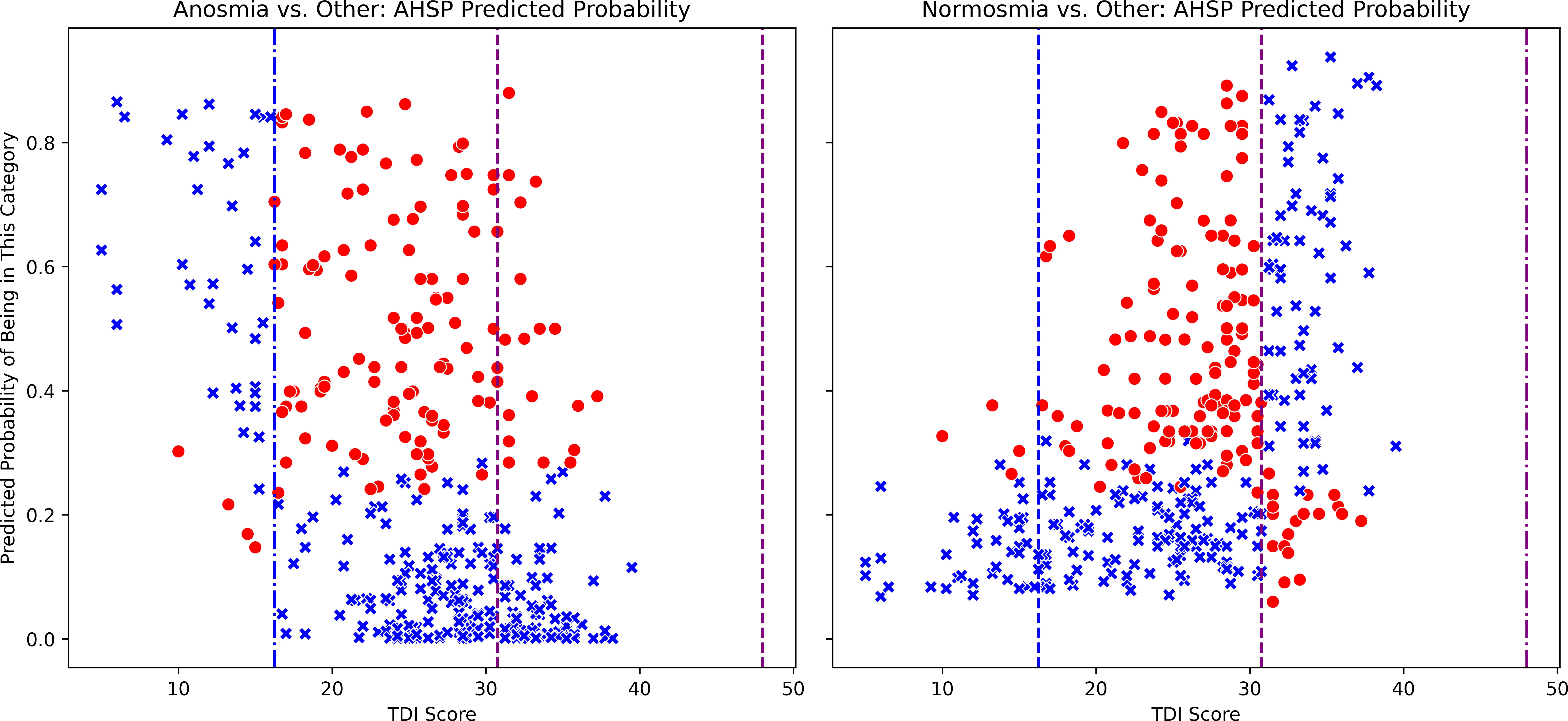


- - 1. AHSP


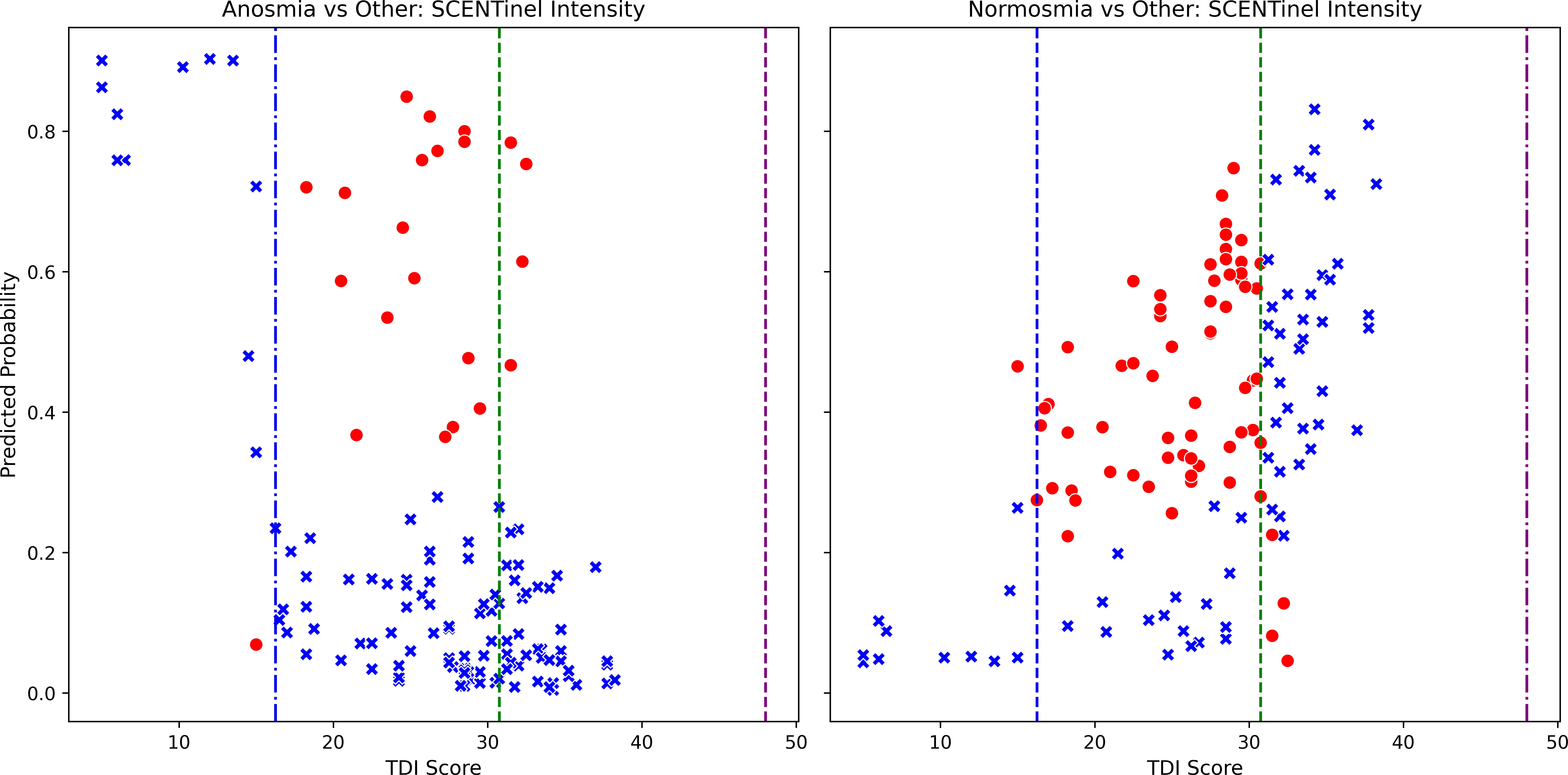


- - 1. SCENTinel Intensity


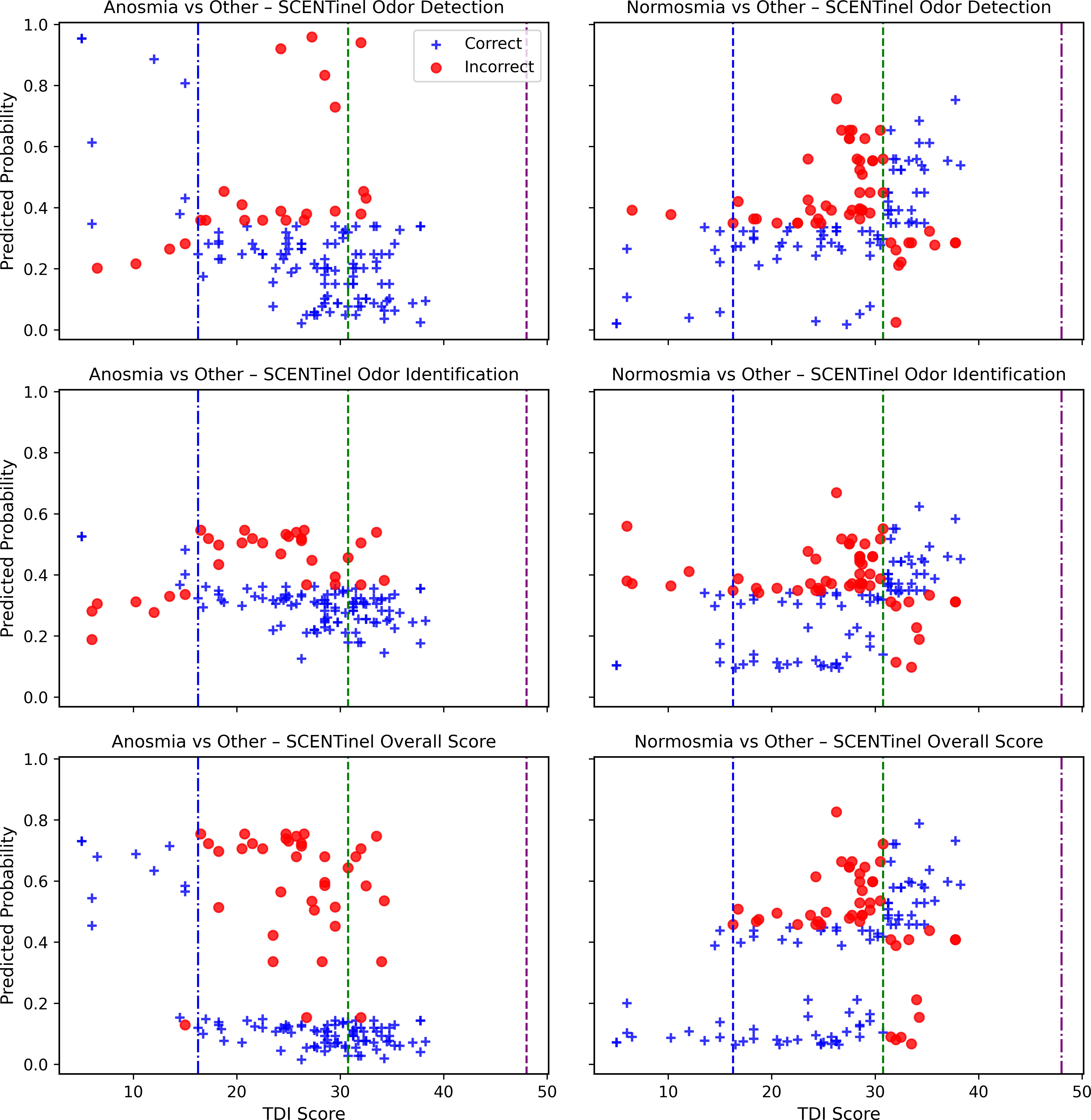


- - 1. SCENTinel Binary

Figure 1: Predicted probabilities for Anosmia, and Normosmia (vs. Other) across TDI scores, using threshold-based classification. Blue points indicate correctly classified individuals, red points indicate misclassifications. Vertical lines represent TDI cutoffs for anosmia (16.25), hyposmia (30.75), and normosmia (48.0). Note that each plot reflects a one-vs-rest binary classification; blue points beyond a category’s diagnostic range represent correct rejections (e.g., correctly not classified as anosmic).

*Appendix A.3. Confusion Matrix*

Table 2: Confusion matrices for each test across diagnostic categories.

| **Test** | **Anosmia vs. Other** | **Normosmia vs. Other** |
| --- | --- | --- |
| **VAS** | [[229, 79], [8, 32]] | [[213, 112], [4, 37]] |
| **AHSP** | [[262, 46], [7, 28]] | [[161, 97], [20, 65]] |
| **GCCR-Check** | [[272, 47], [7, 28]] | [[190, 90], [17, 58]] |
| **SCENT-O** | [[101, 21], [1, 11]] | [[229, 79], [8, 32]] |
| SCENT-D | [[86, 36], [1, 11]] | [[191, 72], [8, 37]] |
| SCENT-Int | [[101, 21], [1, 11]] | [[229, 79], [8, 32]] |
| SCENT-I | [[88, 36], [7, 51]] | [[160, 68], [20, 56]] |

*Note:* The confusion matrices represent the distribution of [[True Negative, False Positive], [False Negative, True Positive]] for binary comparisons against anosmia and normosmia.

*Appendix A.4. Pairwise Comparison*

| Table 3: Pairwise DeLong comparison of test AUCs by category. | | | | Statistically significant p-adjusted values are | | | | |
| --- | --- | --- | --- | --- | --- | --- | --- | --- |
| shown in bold. | |  |  |  |  |  |  |  |
| Category | Comparison | AUC1 | SE AUC1 | AUC2 | SE AUC2 | Z-Score | p-value | p-adjusted |
|  | **VAS vs GCCR-Check** | 0.83 | 0.01 | 0.88 | 0.01 | -4.37 | *<* 0*.*001 | *<* 0*.*001 |
|  | **VAS vs SCENT-O** | 0.83 | 0.01 | 0.82 | 0.01 | 0.15 | 0.883 | 0.928 |
|  | **VAS vs SCENT-Int** | 0.83 | 0.01 | 0.91 | 0.01 | -4.54 | *<* 0*.*001 | *<* 0*.*001 |
|  | **VAS vs SCENT-D** | 0.83 | 0.01 | 0.81 | 0.02 | 2.41 | 0.016 | **0.041** |
|  | **VAS vs SCENT-I** | 0.83 | 0.01 | 0.60 | 0.02 | 6.60 | *<* 0*.*001 | *<* 0*.*001 |
|  | **AHSP vs VAS** | 0.83 | 0.01 | 0.83 | 0.01 | 0.01 | 0.996 | 0.996 |
|  | **AHSP vs GCCR-Check** | 0.83 | 0.01 | 0.88 | 0.01 | -3.77 | *<* 0*.*001 | **0.001** |
|  | **AHSP vs SCENT-O** | 0.83 | 0.01 | 0.82 | 0.01 | 0.55 | 0.584 | 0.721 |
|  | **AHSP vs SCENT-Int** | 0.83 | 0.01 | 0.91 | 0.01 | -4.42 | *<* 0*.*001 | *<* 0*.*001 |
| Anosmia | **AHSP vs SCENT-D**  **AHSP vs SCENT-I** | 0.83  0.83 | 0.01  0.01 | 0.81  0.60 | 0.02  0.02 | 2.95  7.29 | 0.003  *<* 0*.*001 | **0.012**  *<* 0*.*001 |
|  | **GCCR-Check vs SCENT-O** | 0.88 | 0.01 | 0.82 | 0.01 | 2.52 | 0.012 | **0.034** |
|  | **GCCR-Check vs SCENT-Int** | 0.88 | 0.01 | 0.91 | 0.01 | -1.93 | 0.053 | 0.109 |
|  | **GCCR-Check vs SCENT-D** | 0.88 | 0.01 | 0.81 | 0.02 | 5.28 | *<* 0*.*001 | *<* 0*.*001 |
|  | **GCCR-Check vs SCENT-I** | 0.88 | 0.01 | 0.60 | 0.02 | 6.64 | *<* 0*.*001 | *<* 0*.*001 |
|  | **SCENT-D vs SCENT-O** | 0.81 | 0.02 | 0.82 | 0.02 | 0.68 | 0.500 | 0.679 |
|  | **SCENT-D vs SCENT-I** | 0.81 | 0.02 | 0.60 | 0.03 | 6.33 | *<* 0*.*001 | *<* 0*.*001 |
|  | **SCENT-Int vs SCENT-O** | 0.91 | 0.01 | 0.82 | 0.02 | 3.84 | *<* 0*.*001 | **0.001** |
|  | **SCENT-Int vs SCENT-D** | 0.91 | 0.01 | 0.81 | 0.02 | 3.17 | 0.002 | **0.006** |
|  | **SCENT-Int vs SCENT-I** | 0.91 | 0.01 | 0.60 | 0.03 | 9.48 | *<* 0*.*001 | *<* 0*.*001 |
|  | **SCENT-I vs SCENT-O** | 0.60 | 0.03 | 0.82 | 0.02 | -5.65 | *<* 0*.*001 | *<* 0*.*001 |
|  | **VAS vs GCCR-Check** | 0.74 | 0.01 | 0.72 | 0.01 | 0.60 | 0.546 | 0.703 |
|  | **VAS vs SCENT-O** | 0.74 | 0.02 | 0.69 | 0.02 | 2.77 | 0.006 | **0.018** |
|  | **VAS vs SCENT-Int** | 0.74 | 0.02 | 0.68 | 0.02 | 1.75 | 0.080 | 0.153 |
|  | **VAS vs SCENT-D** | 0.74 | 0.02 | 0.64 | 0.02 | 3.39 | 0.001 | **0.003** |
|  | **VAS vs SCENT-I** | 0.74 | 0.02 | 0.65 | 0.02 | 3.49 | *<* 0*.*001 | **0.002** |
|  | **AHSP vs VAS** | 0.72 | 0.01 | 0.74 | 0.01 | -0.63 | 0.532 | 0.698 |
|  | **AHSP vs GCCR-Check** | 0.72 | 0.01 | 0.72 | 0.01 | -0.16 | 0.874 | 0.928 |
|  | **AHSP vs SCENT-O** | 0.72 | 0.02 | 0.69 | 0.02 | 2.08 | 0.038 | 0.082 |
|  | **AHSP vs SCENT-Int** | 0.72 | 0.02 | 0.68 | 0.02 | 1.23 | 0.217 | 0.326 |
|  | **AHSP vs SCENT-Dn** | 0.72 | 0.02 | 0.64 | 0.02 | 2.73 | 0.006 | **0.020** |
| Normosmia | **AHSP vs SCENT-I** | 0.72 | 0.02 | 0.65 | 0.02 | 2.80 | 0.005 | **0.018** |
|  | **GCCR-Check vs SCENT-O** | 0.72 | 0.02 | 0.69 | 0.02 | 2.00 | 0.045 | 0.095 |
|  | **GCCR-Check vs SCENT-Int** | 0.72 | 0.02 | 0.68 | 0.02 | 1.56 | 0.120 | 0.199 |
|  | **GCCR-Check vs SCENT-D** | 0.72 | 0.02 | 0.64 | 0.02 | 2.69 | 0.007 | **0.022** |
|  | **GCCR-Check vs SCENT-I** | 0.72 | 0.02 | 0.65 | 0.02 | 2.40 | 0.016 | **0.041** |
|  | **SCENT-D vs SCENT-O** | 0.64 | 0.03 | 0.69 | 0.03 | -0.97 | 0.331 | 0.473 |
|  | **SCENT-D vs SCENT-I** | 0.64 | 0.03 | 0.65 | 0.03 | -0.41 | 0.684 | 0.824 |
|  | **SCENT-Int vs SCENT-O** | 0.68 | 0.03 | 0.69 | 0.03 | 0.69 | 0.492 | 0.679 |
|  | **SCENT-Int vs SCENT-D** | 0.68 | 0.03 | 0.64 | 0.03 | 1.66 | 0.097 | 0.168 |
|  | **SCENT-Int vs SCENT-I** | 0.68 | 0.03 | 0.65 | 0.03 | 1.25 | 0.210 | 0.323 |
|  | **SCENT-I vs SCENT-O** | 0.65 | 0.03 | 0.69 | 0.03 | -0.57 | 0.571 | 0.720 |
