# Supplementary material for: Evaluating methods for quantitative olfactory assessment: a comparative longitudinal analysis of Sniffin’ Sticks and alternative tools
Source: Chem Senses. 2026 May 6;51:bjag012. doi: 10.1093/chemse/bjag012 (PMC13214561; doi:10.1093/chemse/bjag012)
Supplement: bjag012_Supplementary_Data [file bjag012_supplementary_data.zip › Supplementary Figure Legends.docx]

**Supplementary Figure A.3.** Predicted probabilities for anosmia and normosmia (vs.

Other) across TDI scores, using threshold-based classification, for (A) VAS, (B) GCCR-Check,

(C) AHSP, (D) SCENTinel Intensity, and (E) SCENTinel Binary. Blue points indicate correctly

classified individuals; red points indicate misclassifications. Vertical lines represent TDI cutoffs

for anosmia (16.25), hyposmia (30.75), and normosmia (48.0).

Alt text: Five scatter plots arranged vertically, one per olfactory test. In each plot, predicted

probability is shown on the y-axis and TDI score on the x-axis. Blue points represent correct

classifications and red points represent misclassifications. Three vertical dashed lines mark TDI

cutoffs at 16.25, 30.75, and 48.0. Panels show VAS, GCCR-Check, AHSP, SCENTinel Intensity,

and SCENTinel Binary from top to bottom.
