# Supplementary material for: Evaluating methods for quantitative olfactory assessment: a comparative longitudinal analysis of Sniffin’ Sticks and alternative tools
Source: Chem Senses. 2026 May 6;51:bjag012. doi: 10.1093/chemse/bjag012 (PMC13214561; doi:10.1093/chemse/bjag012)
Supplement: bjag012_Supplementary_Data [file bjag012_supplementary_data.zip › Supplementary Table Legends.docx]

**Supplementary Table A.7.** Correlation between alternative tests and TDI scores using raw data and GEE. Correlation coefficients were obtained using GEE models with an exchangeable correlation structure.

**Supplementary Table A.8.** Confusion matrices for each test across diagnostic categories (anosmia vs. Other; normosmia vs. Other), showing the distribution of true negatives, false positives, false negatives, and true positives.

**Supplementary Table A.9.** Pairwise DeLong comparison of test AUCs by category. Statistically significant p-adjusted values are shown in bold.
