# Supplementary material for: Evaluating methods for quantitative olfactory assessment: a comparative longitudinal analysis of Sniffin’ Sticks and alternative tools
Source: Chem Senses. 2026 May 6;51:bjag012. doi: 10.1093/chemse/bjag012 (PMC13214561; doi:10.1093/chemse/bjag012)
Supplement: bjag012_Supplementary_Data [file bjag012_supplementary_data.zip › supplementary tables.docx]

Table A.7: Correlation between alternative tests and TDI using raw data and GEE

| **Test** | **Correlation Coefficient** | **SE** | **95% CI** | **p-value** |
| --- | --- | --- | --- | --- |
| VAS | 0.08 | 0.01 | [0.060, 0.109] | <0.001 |
| AHSP | 0.52 | 0.08 | [0.362, 0.679] | <0.001 |
| GCCR-Check | 0.09 | 0.01 | [0.068, 0.123] | <0.001 |
| SCENT-O | - | - | - | <0.001* |

*Note:* Correlation coefficients were obtained using GEE models with an exchangeable correlation structure. SE = Standard Error, CI = Confidence Interval.

* Direct correlation coefficient was not computed for SCENTinel due to data structure differences.

Table A.8: Confusion Matrices for each test Across Diagnostic Categories.

| **Test** | **Anosmia vs. Other** | **Normosmia vs. Other** |
| --- | --- | --- |
| **VAS** | [[229, 79], [8, 32]] | [[213, 112], [4, 37]] |
| **AHSP** | [[262, 46], [7, 28]] | [[161, 97], [20, 65]] |
| **GCCR-Check** | [[272, 47], [7, 28]] | [[190, 90], [17, 58]] |
| **SCENT-O** | [[101, 21], [1, 11]] | [[229, 79], [8, 32]] |
| SCENT-D | [[86, 36], [1, 11]] | [[191, 72], [8, 37]] |
| SCENT-Int | [[101, 21], [1, 11]] | [[229, 79], [8, 32]] |
| SCENT-I | [[88, 36], [7, 51]] | [[160, 68], [20, 56]] |

Note: The confusion matrices represent the distribution of [True Negative, False Positives], [False Negatives, and True Positives] across the different diagnostic categories (Anosmia, Hyposmia, Normosmia) for each test.

Table A.9: Pairwise DeLong comparison of test AUCs by category. Statistically significant comparisons (after FDR correction) are shown in bold.

| Category | Comparison | AUC1 | | SE_AUC1 | | AUC2 | | SE_AUC2 | | Z-Score | | p-value | | p-adjusted |
| --- | --- | --- | --- | --- | --- | --- | --- | --- | --- | --- | --- | --- | --- | --- |
| **Anosmia vs. Other** | **VAS vs GCCR-Check** | 0.83 | | 0.01 | | 0.88 | | 0.01 | | -4.37 | | <0.001 | | <0.001 |
|  | **VAS vs SCENT-O** | 0.83 | | 0.01 | | 0.82 | | 0.01 | | 0.15 | | 0.883 | | 0.928 |
|  | **VAS vs SCENT-Int** | 0.83 | | 0.01 | | 0.91 | | 0.01 | | -4.54 | | <0.001 | | <0.001 |
|  | **VAS vs SCENT-D** | 0.83 | | 0.01 | | 0.81 | | 0.02 | | 2.41 | | 0.016 | | **0.041** |
|  | **VAS vs SCENT-I** | 0.83 | | 0.01 | | 0.60 | | 0.02 | | 6.60 | | <0.001 | | <0.001 |
|  | **AHSP vs VAS** | 0.83 | | 0.01 | | 0.83 | | 0.01 | | 0.01 | | 0.996 | | 0.996 |
|  | **AHSP vs GCCR-Check** | 0.83 | | 0.01 | | 0.88 | | 0.01 | | -3.77 | | <0.001 | | **0.001** |
|  | **AHSP vs SCENT-O** | 0.83 | | 0.01 | | 0.82 | | 0.01 | | 0.55 | | 0.584 | | 0.721 |
|  | **AHSP vs SCENT-Int** | 0.83 | | 0.01 | | 0.91 | | 0.01 | | -4.42 | | <0.001 | | <0.001 |
|  | **AHSP vs SCENT-D** | 0.83 | | 0.01 | | 0.81 | | 0.02 | | 2.95 | | 0.003 | | **0.012** |
|  | **AHSP vs SCENT-I** | 0.83 | | 0.01 | | 0.60 | | 0.02 | | 7.29 | | <0.001 | | <0.001 |
|  | **GCCR-Check vs SCENT-O** | 0.88 | | 0.01 | | 0.82 | | 0.01 | | 2.52 | | 0.012 | | **0.034** |
|  | **GCCR-Check vs SCENT-Int** | 0.88 | | 0.01 | | 0.91 | | 0.01 | | -1.93 | | 0.053 | | 0.109 |
|  | **GCCR-Check vs SCENT-D** | 0.88 | | 0.01 | | 0.81 | | 0.02 | | 5.28 | | <0.001 | | <0.001 |
|  | **GCCR-Check vs SCENT-I** | 0.88 | | 0.01 | | 0.60 | | 0.02 | | 6.64 | | <0.001 | | <0.001 |
|  | **SCENT-D vs SCENT-O** | 0.81 | | 0.02 | | 0.82 | | 0.02 | | 0.68 | | 0.500 | | 0.679 |
|  | **SCENT-D vs SCENT-I** | 0.81 | | 0.02 | | 0.60 | | 0.03 | | 6.33 | | <0.001 | | <0.001 |
|  | **SCENT-Int vs SCENT-O** | 0.91 | | 0.01 | | 0.82 | | 0.02 | | 3.84 | | <0.001 | | **0.001** |
|  | **SCENT-Int vs SCENT-D** | 0.91 | | 0.01 | | 0.81 | | 0.02 | | 3.17 | | 0.002 | | **0.006** |
|  | **SCENT-Int vs SCENT-I** | 0.91 | | 0.01 | | 0.60 | | 0.03 | | 9.48 | | <0.001 | | <0.001 |
|  | **SCENT-I vs SCENT-O** | 0.60 | | 0.03 | | 0.82 | | 0.02 | | -5.65 | | <0.001 | | <0.001 |
| **Normosmia vs. Other** | **VAS vs GCCR-Check** | 0.74 | | 0.01 | | 0.72 | | 0.01 | | 0.60 | | 0.546 | | 0.703 |
|  | **VAS vs SCENT-O** | 0.74 | | 0.02 | | 0.69 | | 0.02 | | 2.77 | | 0.006 | | **0.018** |
|  | **VAS vs SCENT-Int** | 0.74 | | 0.02 | | 0.68 | | 0.02 | | 1.75 | | 0.080 | | 0.153 |
|  | **VAS vs SCENT-D** | 0.74 | | 0.02 | | 0.64 | | 0.02 | | 3.39 | | 0.001 | | **0.003** |
|  | **VAS vs SCENT-I** | 0.74 | | 0.02 | | 0.65 | | 0.02 | | 3.49 | | <0.001 | | **0.002** |
|  | **AHSP vs VAS** | 0.72 | | 0.01 | | 0.74 | | 0.01 | | -0.63 | | 0.532 | | 0.698 |
|  | **AHSP vs GCCR-Check** | 0.72 | | 0.01 | | 0.72 | | 0.01 | | -0.16 | | 0.874 | | 0.928 |
|  | **AHSP vs SCENT-O** | 0.72 | | 0.02 | | 0.69 | | 0.02 | | 2.08 | | 0.038 | | 0.082 |
|  | **AHSP vs SCENT-Int** | 0.72 | | 0.02 | | 0.68 | | 0.02 | | 1.23 | | 0.217 | | 0.326 |
|  | **AHSP vs SCENT-D** | 0.72 | | 0.02 | | 0.64 | | 0.02 | | 2.73 | | 0.006 | | **0.020** |
|  | **AHSP vs SCENT-I** | 0.72 | | 0.02 | | 0.65 | | 0.02 | | 2.80 | | 0.005 | | **0.018** |
|  | **GCCR-Check vs SCENT-O** | 0.72 | | 0.02 | | 0.69 | | 0.02 | | 2.00 | | 0.045 | | 0.095 |
|  | **GCCR-Check vs SCENT-Int** | 0.72 | | 0.02 | | 0.68 | | 0.02 | | 1.56 | | 0.120 | | 0.199 |
|  | **GCCR-Check vs SCENT-D** | 0.72 | | 0.02 | | 0.64 | | 0.02 | | 2.69 | | 0.007 | | **0.022** |
|  | **GCCR-Check vs SCENT-I** | 0.72 | | 0.02 | | 0.65 | | 0.02 | | 2.40 | | 0.016 | | **0.041** |
|  | **SCENT-D vs SCENT-O** | 0.64 | | 0.03 | | 0.69 | | 0.03 | | -0.97 | | 0.331 | | 0.473 |
|  | **SCENT-D vs SCENT-I** | 0.64 | | 0.03 | | 0.65 | | 0.03 | | -0.41 | | 0.684 | | 0.824 |
|  | **SCENT-Int vs SCENT-O** | 0.68 | | 0.03 | | 0.69 | | 0.03 | | 0.69 | | 0.492 | | 0.679 |
|  | **SCENT-Int vs SCENT-D** | 0.68 | 0.03 | | 0.64 | | 0.03 | | 1.66 | | 0.097 | | 0.168 | |
|  | **SCENT-Int vs SCENT-I** | 0.68 | 0.03 | | 0.65 | | 0.03 | | 1.25 | | 0.210 | | 0.323 | |
|  | **SCENT-I vs SCENT-O** | 0.65 | 0.03 | | 0.69 | | 0.03 | | -0.57 | | 0.571 | | 0.720 | |
